# Marginal quality of ceramic inlays after three different instrumental cavity preparation methods of the proximal boxes

**DOI:** 10.1007/s00784-018-2492-0

**Published:** 2018-06-04

**Authors:** Ella A. Naumova, Fabian Schiml, Wolfgang H. Arnold, Andree Piwowarczyk

**Affiliations:** 1 Department of Biological and Material Sciences in Dentistry, School of Dentistry, Faculty of Health, Witten/Herdecke University, Alfred-Herrhausen-Strasse 44, 58455 Witten, Germany; 2 Department of Prosthodontics, School of Dentistry, Faculty of Health, Witten/Herdecke University, Alfred-Herrhausen-Strasse 44, 58455 Witten, Germany

**Keywords:** Surface roughness, Microleakage, Marginal gap, Margin quality, Ceramic inlays, Finishing methods, Hydrothermal wear

## Abstract

**Objectives:**

The marginal quality of ceramic inlays was evaluated after the use of three different instrumental finishing methods in mesio-occluso-distal (mod) cavity boxes in vitro after hydrothermal loading (HTL).

**Materials and methods:**

Caries-free human molars were divided into three groups. Mod-cavities were conventionally prepared. Box finishing was performed in every group with rotating (RI), sonic (SI), or ultrasonic (USI) instruments. Surface roughness was examined. Twelve mod-cavities remained untreated. Continuous margin quality was evaluated with scanning electron microscopy (SEM). Ceramic inlays were cemented into cavities. After HTL microleakage, marginal and absolute marginal gaps were examined. All data were analyzed statistically.

**Results:**

Significant differences were found, between cavity surface roughness of RI and SI groups, the RI and USI groups, but not between microleakage, marginal, absolute marginal gaps after HTL and in proximal marginal quality. No correlations between microleakage and marginal gaps nor between microleakage and surface roughness were found.

**Conclusion:**

Mod-cavity proximal box finishing with SI or USI resulted in a higher surface roughness than the use of RI. The type of the finishing method did not influence the marginal quality of ceramic inlays. For the mod-cavity finishing, the use of SI and USI could be an alternative instrumental method to conventional RI methods with a lower risk of iatrogenic damage of the adjacent teeth.

**Clinical relevance:**

This study allows the practitioner to better determine the proper indications and limitations of the sonic and ultrasonic instruments for mod-cavity proximal box finishing.

## Introduction

Qualitative cavity preparation without discomfort is very important in restorative dentistry [1]. The restoration geometry, design considerations, preparation methods, and loading conditions are key factors for the long-term success of dental restorations [2–8]. For tooth-colored ceramic inlays in esthetic dentistry, the long-term success is an actual problem [7]. Accordingly, information and discussion about the applications and effects of the different instrumental methods of cavity preparation [1, 9], technical requirements, and the effective preparation of dental tissues [7] are in demand.

According to the concept of “minimally hazardous dentistry” [7, 10], technical details about the application of different instruments for cavity preparation are clinically relevant because side effects, such as border defects and very irregular surfaces, especially in proximal box cavities [9]; cracks, especially in the enamel [1]; and iatrogenic damage to adjacent teeth [11–13], may occur. This mechanical damage may result in possible biological complications, such as tooth sensitivity and bacterial leakage [7]. Different types of instrumental methods for dental cavity preparation are currently in use: conventional-rotating [9], laser [1, 14, 15], sonic [9], or ultrasonic preparation [16, 17]. Mechanical damage of the teeth is more likely to occur with the use of rotating instruments [9]. Ultrasonic instruments are widely used in dentistry because of better efficiency, visualization, operative convenience, precise cutting ability [17], and success in accessing difficult areas on the preparation margin [16].

Molar esthetic restoration is in demand [18]. Ceramic inlays are indicated for tooth restoration in extended mod-cavities with a loss of proximal contacts [19]. The marginal quality of ceramic inlays, especially the quality of the proximal margins, is very important for providing highly esthetic, long-lasting, plaque-resistant restorations [2, 20, 21]. The presence of surface irregularities in the inlay proximal area can increase plaque formation, gingival irritation, recurrent caries, abrasiveness, wear kinetics, staining, and tactile perception by the patient [20, 22–24]. Marginal quality is of clinical importance for the process of bacterial retention [22, 25]. Therefore, the possible influence of different types of dental restorations on microleakage and marginal adaptation should be demonstrated with the use of different methods for cavity preparation [1, 2]. However, only limited information is available about the proximal margin quality of the ceramic inlays in molar teeth after mesio-occluso-distal cavity proximal box finishing with sonic and ultrasonic instruments after hydrothermal loading (HTL) in vitro [9, 21].

The marginal quality of the ceramic inlay approximal area is of clinical importance because the ceramic inlay approximal area is located in the interdental space, and tooth brushing in this area is restricted [16, 26].

The aim of this in vitro study is to evaluate the influence of different alternative methods of shaping and finishing mod-cavity proximal boxes on the adaptation of ceramic inlays to the tooth morphology and bonding quality.

The null hypothesis for the present study was that three different finishing methods of mod-cavities in human molars would not differ in their effects on the surface roughness and border quality of the proximal boxes, microleakage, and marginal gap of the ceramic inlays within the enamel.

## Materials and methods

### Tooth collection

**Fig. 1:**
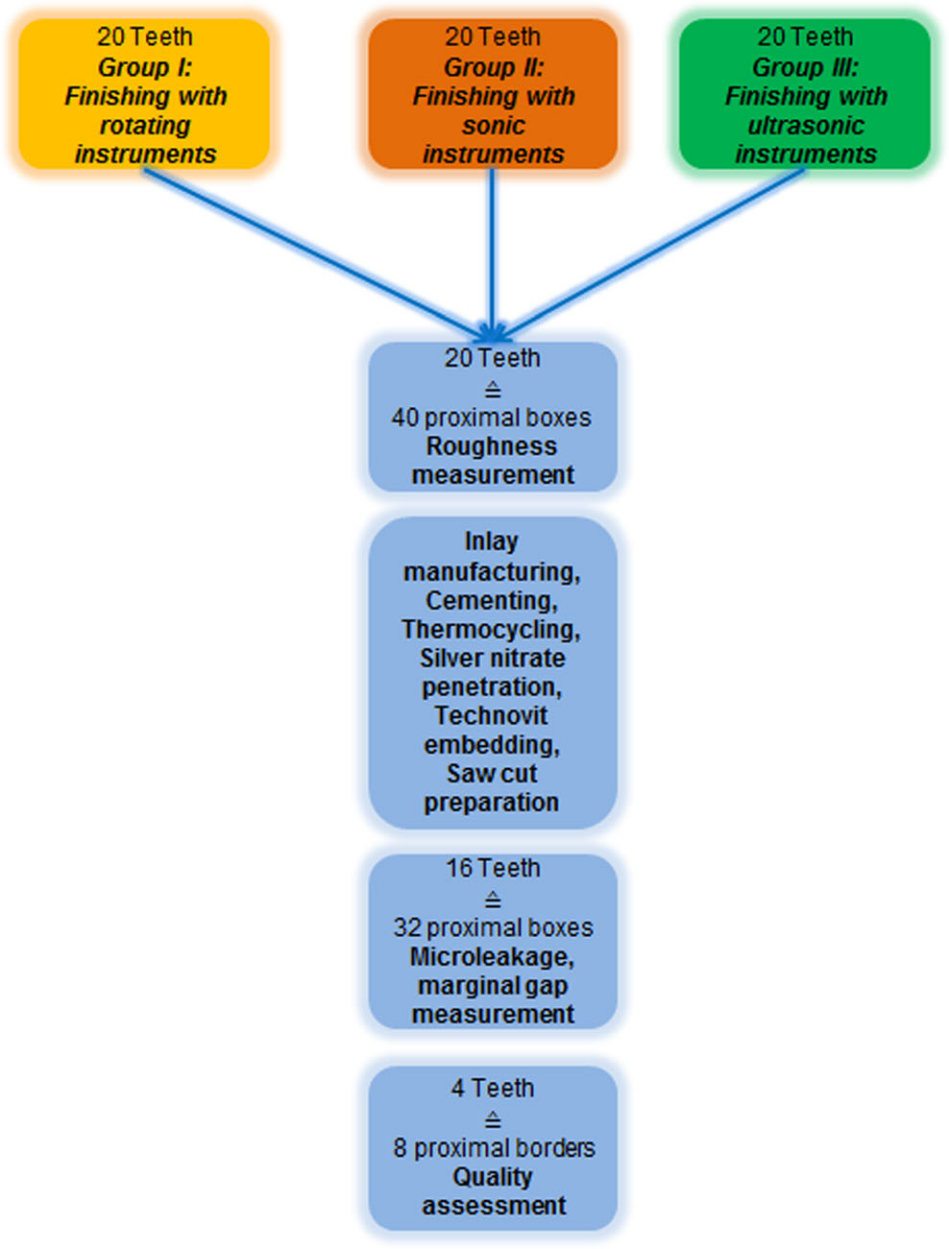
Set-up of the present study

Tooth collection was approved by the ethical committee of Witten/Herdecke University (permission 116/2013). For this study, a total of 60 extracted human molar teeth were collected. They were caries-free, lacked dental calculus, and had completed root growth, comparable sizes, and absolute integrity. Immediately after extraction, the teeth were stored in 0.9% NaCl containing 0.1% thymol at room temperature (maximum 1–3 months) until use. Tissue remnants on the teeth surface were removed with a universal scaler. The teeth were randomly distributed into three groups (Fig. 1).

### Cavity preparation

**Fig. 2:**
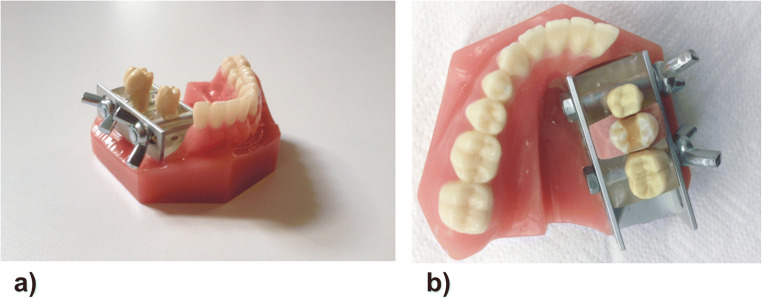
The specially constructed original lower jaw model: **a** without the experimental teeth, **b** with the experimental teeth with the prepared mod-cavity for the full ceramic inlay

For the realistic simulation of tooth preparation, a specially designed original lower jaw model was constructed for precise positioning and stable fixation of the experimental teeth (Fig. 2). Initially, the roots of the experimental teeth were coated with condensation-K-Silikon (HLW Dental, Wernberg-Köblitz, Germany). Then, the experimental teeth were placed in the U-profile (the row) of the lower jaw between teeth 45 and 47 (Frasaco GmbH, Tettnang, Germany) and aligned along the occlusion. Then, teeth 45 and 47 were moved until the proximal contacts from both sides of the experimental tooth were reached. Thereafter, the original lower jaw model with the experimental tooth was placed and fixed in the head model (Frasaco GmbH, Tettnang, Germany) with the face mask P-6 GM (Frasaco GmbH, Tettnang, Germany) and the opposing upper jaw ANKA-4 (Frasaco GmbH, Tettnang, Germany).

All specimen preparations were performed by the same operator and calibrated prior to the study by one of the authors to avoid inter-examiner differences.

For the preparation, the AG ceramics guidelines of Ahlers et al. 2009 [27] for a ceramic inlay with a mod-cavity were followed. The following geometrical parameters for the mod-cavity were kept: a diverging angle of approximately 6°, a minimal depth of the occlusal box in the deepest area of the fissure of approximately 1.5 mm, a minimal width of the isthmus of approximately 1.5 mm, a cavity surface angle of approximately 90°, and a planar occlusal and proximal cavity floor. The cervical margin preparation was located in enamel with a maximum dimension of 1.5 mm above the enamel-cementum junction.

The dimensions of the mod-cavities were maintained with the preparation instruments with defined conicity and laser depth markings and were finally controlled with the CP-15 UNC periodontometer (Hu-Friedy Mfg. Co., LLC, Tuttlingen, Germany). The transitions within the mod-cavity were rounded off, and the proximal contacts to the adjacent tooth were completely separated.

The mod-cavity preparation was performed in three steps: (1) initial whole mod-cavity shaping in all groups (*n* = 60), (2) mod-cavity proximal box finishing within every three groups (*n* = 20), and (3) rounding and smoothing all sharp edges inside of the mod-cavity in all groups (*n* = 60). Initial whole cavity shaping for 60 teeth was performed in all groups using the same method with the Synea Vision WK-99 LT handpiece (W & H Germany GmbH, Laufen, Germany) under ca. 80% power (ratio 1:5, engine speed 32,000 rpm, speed of the instrument 160,000 rpm) and diamond instruments. Then, a purely occlusal limited cavity along the main fissure was produced with the 959KRD 314,018 non-ringed diamond conical grinder (Komet, Lemgo, Germany) (grain size ca. 100 μm, laser marking at 2 and 4 mm, conicity ca. 2°). After this, the proximal box to the dissolution of the basal contact point was created with a 6847KRD 314 016 green ringed conical diamond grinder (Komet, Lemgo, Germany) (grain size ca. 125 μm, laser marking at 2 and 4 mm, conicity ca. 2°). This was done with the protection of the adjacent tooth with steel-band matrices, which were fixed with a wooden wedge. The occlusal box was smoothed with the red-ringed conical diamond instrument 8959KR 314 018 (Komet, Lemgo, Germany) (grain size ca. 30–45 μm, conicity ca. 2°). After the initial whole mod-cavity shaping of five teeth, the used instruments were replaced with new ones. This rule was also applied to subsequent proximal box finishing within groups.

Proximal box finishing was performed with three different methods and instruments in the three groups.

#### Proximal box finishing in group 1

Proximal box finishing in group 1 was performed with the red high-speed angle Synea Vision WK-99 LT handpiece (W & H Germany GmbH, Laufen, Germany) under 10–20% power (ratio 1:5, engine speed 4000–8.000 rpm, speed of the instrument 20,000–40,000 rpm) and the rotating red-ringed conical diamond instrument 8847KR 314 016 (Komet, Lemgo, Germany) (grain size ca. 30–45 μm, conicity ca. 2°); the neighboring teeth were protected by steel-band matrices.

#### Proximal box finishing in group 2

Proximal box finishing in group 2 was performed with the SF1LM sonic handpiece (Komet, Lemgo, Germany) and with SFD7 000 1 (distal) and SFM7 000 1 (mesial) sonic tips (Komet, Lemgo, Germany) (grain size ca. 60 μm, conicity ca. 8°) on power setting 3 for shaping (amplitude of the free axial oscillation: 180–200 μm) and on power setting 1 for finishing (amplitude of the free axial oscillation: 100–140 μm).

#### Proximal box finishing in group 3

Proximal box finishing in group 3 was performed with the ultrasonic Sirona Perioscan drive (Sirona Dental GmbH, Wals, Austria), the ultrasound Periosonic handpiece (Sirona Dental GmbH, Wals, Austria), and the SFD7 000 1 (distal) and SFM7 000 1 (mesial) sonic tips (Komet, Lemgo, Germany) (grain size ca. 60 μm, conicity ca. 8°) at 100% power (32 kHz oscillation frequency).

Because the sonic tips on the tooth-facing surface are grainless, the protection of adjacent teeth with the steel band in groups 2 and 3 was not necessary.

In all groups, all sharp edges present inside the cavity were rounded and smoothed with the 8862 314 010 red-ringed diamond flame (Komet, Lemgo, Germany) (grain size ca. 30–45 μm).

### Inlay production

The prepared teeth were imprinted with Impregum Penta H Duosoft and Impregum Garant L Duosoft (3M, Seefeld, Germany) using a double mixing technique at room temperature.

Then, the ceramic inlays were conventionally modeled, pressed, adjusted, and completed according to standard laboratory procedures.

### Quality control of the ceramic inlays

Prior to cementing, two independent examiners visually inspected the ceramic inlays under a magnifying glass (× 2.5). The entire restoration margin was explicitly controlled in the mesial and distal proximal range. Only clinically acceptable inlays were added to the study. Clinically unacceptable inlays were newly manufactured and controlled again.

### Cementation of the ceramic inlays

The basal surface of the ceramic inlay was etched for 20 s with ceramic etching gel that contained 5% hydrofluoric acid IPS (Ivoclar Vivadent AG, Schaan, Liechtenstein), then rinsed for 20 s with the multifunctional syringe, dried and covered for 60 s with the single-component adhesion primer Monobond Plus (Ivoclar Vivadent AG, Schaan, Liechtenstein). Enamel and dentine in the mod-cavity were etched for 30 and 15 s, respectively, with 37% phosphoric acid (Ivoclar Vivadent AG, Schaan, Liechtenstein), then rinsed for 30 s with the multifunctional syringe, dried and covered for 20 s with Universal Adhesive (Ivoclar Vivadent AG, Schaan, Liechtenstein). Adhesive was blown into a uniformly thin film and cured for 10 s with the Satelec Mini Led SP dental curing light (Acteon Germany GmbH, Mettmann, Germany) at a distance of approximately 1 mm. Then, the basal surface of the inlay was covered with the dual-curing luting composite Variolink esthetic DC (Ivoclar Vivadent AG, Schaan, Liechtenstein) and inserted into the mod-cavity with uniform pressure. The excess composite was removed with a foam pellet. Then, the ceramic inlay was kept in the correct position with a Heidemann-spatula under gentle pressure. The inlay was cured for 10 s with the Satelec Mini Led SP dental curing light (Acteon Germany GmbH, Mettmann, Germany) from the occlusal-mesial and occlusal-distal sides at an angle of approximately 45° to the occlusal plane, at a distance of approximately 1 mm from the tooth. To avoid an oxygen-inhibiting layer, the gap between the inlay and the tooth-hard substance before polymerization was covered with a glyceringel liquid strip (Ivoclar Vivadent AG, Schaan, Liechtenstein).

### Artificial aging of the ceramic inlays

Immediately after cementation, the teeth (*n* = 16 from every group) were subjected to thermocycling in the Thermocycler THE1000 (SD Mechatronics, Feldkirchen-Westerham, Germany) with 5000 cycles in water baths at 5 and 55 °C (resistance time 30 s, dripping time 15 s) to simulate hydrothermal stress for the subsequent microleakage and marginal gap examination.

### Silver nitrate (AgNO_3_) penetration

**Fig. 3:**
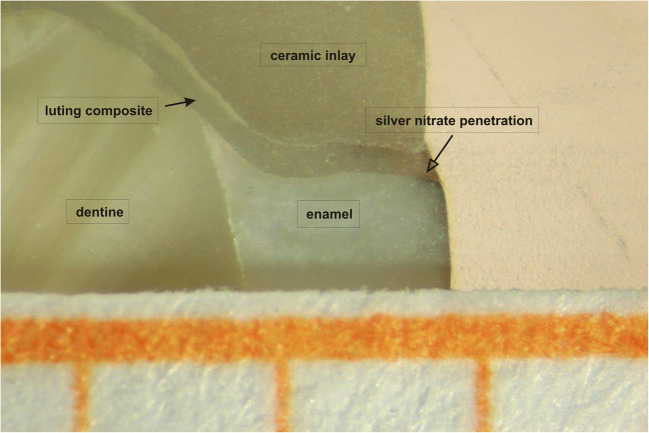
Measurement of the microleakage. The depth of the silver precipitation is marked with an empty arrow, luting composite with a full arrow

After removal from the thermocycler, the teeth were rinsed with distilled water and dried with a paper towel. The dry teeth were sealed with two layers of Pattex Mini Trio superglue (Henkel AG & Co. KGaA, Dusseldorf, Germany). The basal proximal preparation margin and 0.5 mm circular remained unsealed. After drying for 30 min, the teeth were immersed in AgNO_3_ (56.62 g/1 L H_2_O) for 6 h. Subsequently, the teeth were exposed to four illuminants of 100 W for 4 h. After exposure, AgNO_3_ was developed in liquid Periomat Intra developer (Dürr Dental, Bietigheim-Bissingen, Germany). The depth of silver precipitation was measured in the sections (Fig. 3).

### Preparation of sections

All specimens were embedded in Technovit 9100 (Heraeus Kulzer, Werheim, Germany). Then, the specimens were cut in the middle of the sagittal plane through the ceramic inlay with a Leica SP1600 Saw Microtome (Leica Biosystems GmbH, Wetzlar, Germany).

#### Assessment of surface roughness of the proximal box floor

After preparation and before taking an impression, the surface roughness of each proximal box floor was determined with an Alicona Infinite Focus optical measuring system and Alicona IFM 3.2 computer software (Alicona Imaging GmbH, Raaba/Graz, Austria). In three areas of each proximal box floor, five measurements were taken, and the mean roughness (Ra) was determined.

#### Assessment of marginal gap

**Fig. 4:**
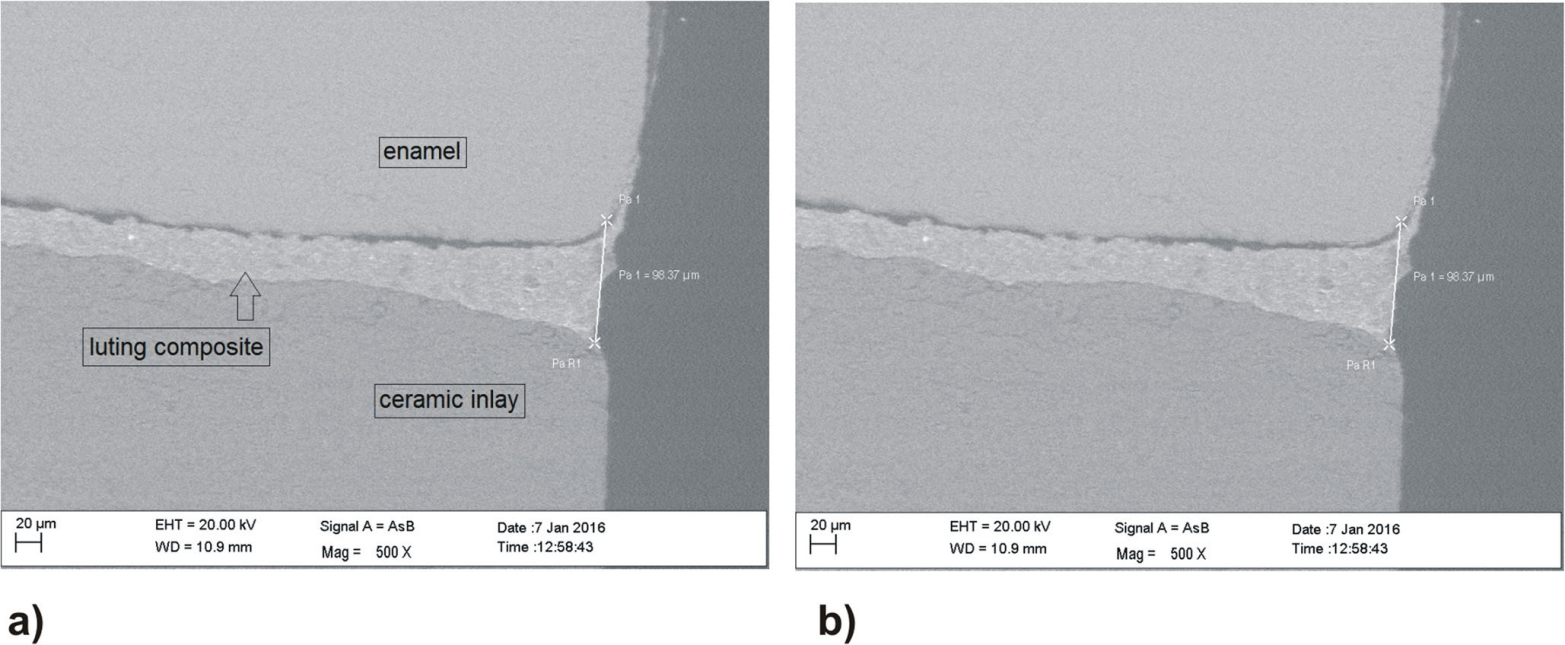
Measurements of the marginal gap (MOP) and absolute marginal gap (aMOP)

The measurement of the marginal gap as defined by Holmes et al. (1989) of the individual specimens was carried out with a scanning electron microscope (Sigma VP, Carl Zeiss AG, Oberkochen, Germany) in low vacuum mode (VP) at 20 Pa at 20 kV and 500-fold magnification. The marginal gap was measured with the “point-to-point-measure” function of the SmartSEM computer software (Carl Zeiss AG, Oberkochen, Germany). The mesial and distal proximal areas of inlays were investigated with respect to the marginal gap and the absolute marginal discrepancy (aMOP gap) [28]. Since the inlay is suitably contoured, the marginal gap and the absolute marginal discrepancy (MOP gap) are equal—in this case, 98.37 μm (Fig. 4).

#### Assessment of microleakage

For the measurement of AgNO_3_ penetration, photographs of the cut samples were taken with a Leica Wild 3ZM stereomicroscope (Leica Camera AG, Wetzlar, Germany). For the computer-assisted measurement of AgNO_3_ penetration, the images were examined with AutoCAD 2011 computer software (Autodesk GmbH, Munich, Germany). The distance between the extreme point of the preparation edge and the central point of AgNO_3_ penetration was measured. These measurements were repeated by an additional independent examiner.

#### Assessment of the ceramic inlay proximal box margin quality

For the evaluation of the ceramic inlay proximal box margin quality, the tooth crowns with the ceramic inlays (*n* = 4 from every group) were cut into two halves in the bucco-oral direction with a diamond disk, sputtered from the proximal side with gold-palladium and investigated with a scanning electron microscope (Sigma VP, Carl Zeiss AG, Oberkochen, Germany) in high vacuum mode at 20-kV acceleration voltage and the secondary electron detector (SE) at 75-fold magnification. Then, the quality of proximal box margin preparation was visually defined, as seen in Table 1 [9, 29]. Finally, the length of the ceramic inlay proximal box margin sections with the different qualities was measured via SEM (× 75) with the “point-to-point-measure” function of the SmartSEM computer software (Carl Zeiss AG, Oberkochen, Germany).

**Table 1:** Definition of the proximal box margin quality

	Quality range	Definition
Clinically acceptable	Quality A1	Margin with regular even course
	Quality A2	Margin with waved course
	Quality A3	Margin irregular, with zig zag course
Clinically unacceptable	Quality B	Margin cannot be defined

### Statistics

Prior to statistical calculations, the normality of the data was checked using the Kolmogorov-Smirnov test. As the data were not normally distributed, non-parametric tests were applied. For the comparison of group differences, the Mann-Whitney *U* test was applied. As multiple comparisons were calculated, the Bonferroni correction of the alpha error resulted in a *p* value of 0.016. Correlations were calculated with the non-parametric Spearman-Rho test. The Statistical Package for Social Sciences program (SPSS, IBM, Amronk, NJ, USA) Vers. 23 was used.

## Results

### Comparison of the proximal box floor surface roughness after using different proximal box finishing methods

**Fig. 5:**
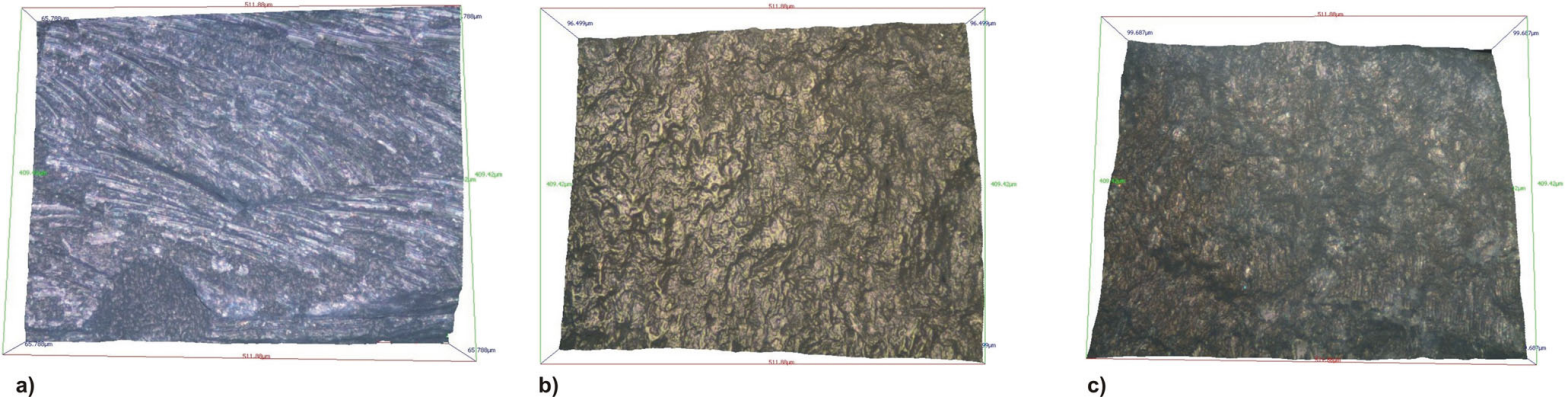
The different patterns of proximal box floor surface roughness after the use of the proximal box finishing methods with three different instruments: **a** rotating, **b** sonic, and **c** ultrasonic

**Fig. 6:**
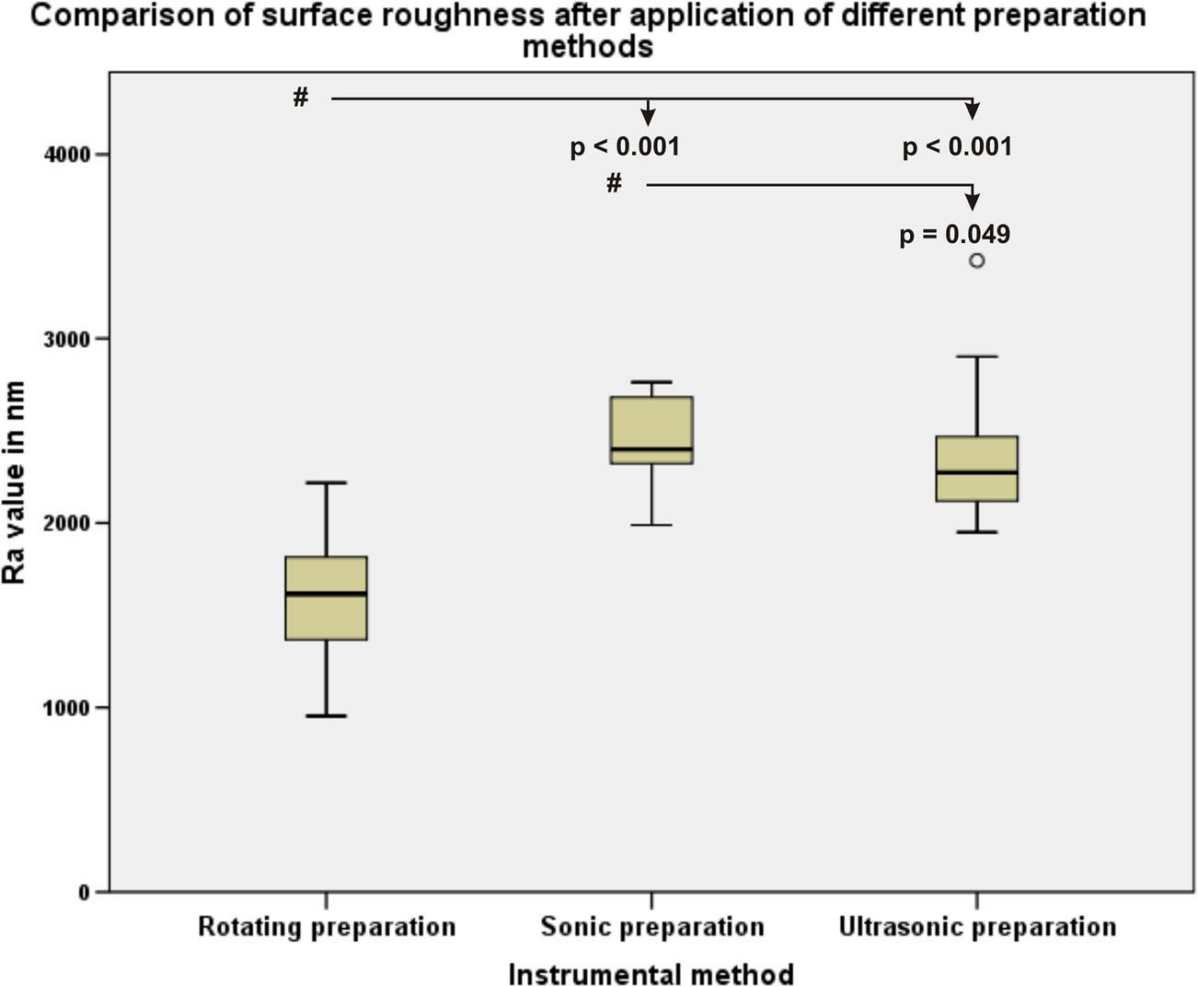
Boxplot of the data distribution of the surface roughness measurements after different preparation methods. The pound symbol indicates the comparisons among data

Microscopically qualitative differences in the surface texture were found after preparation with the different instrumental methods (Fig. 5). A significant increase in the surface roughness of the proximal box floor was found in samples that underwent rotating preparation and sonic preparation and ultrasonic preparation (*p* < 0.001). No significant differences were found in the surface roughness of samples subjected to sonic and ultrasonic preparation (*p* = 0.016) (Fig. 6).

### Comparison of the ceramic inlay proximal microleakage after using the different proximal box finishing methods

**Fig. 7:**
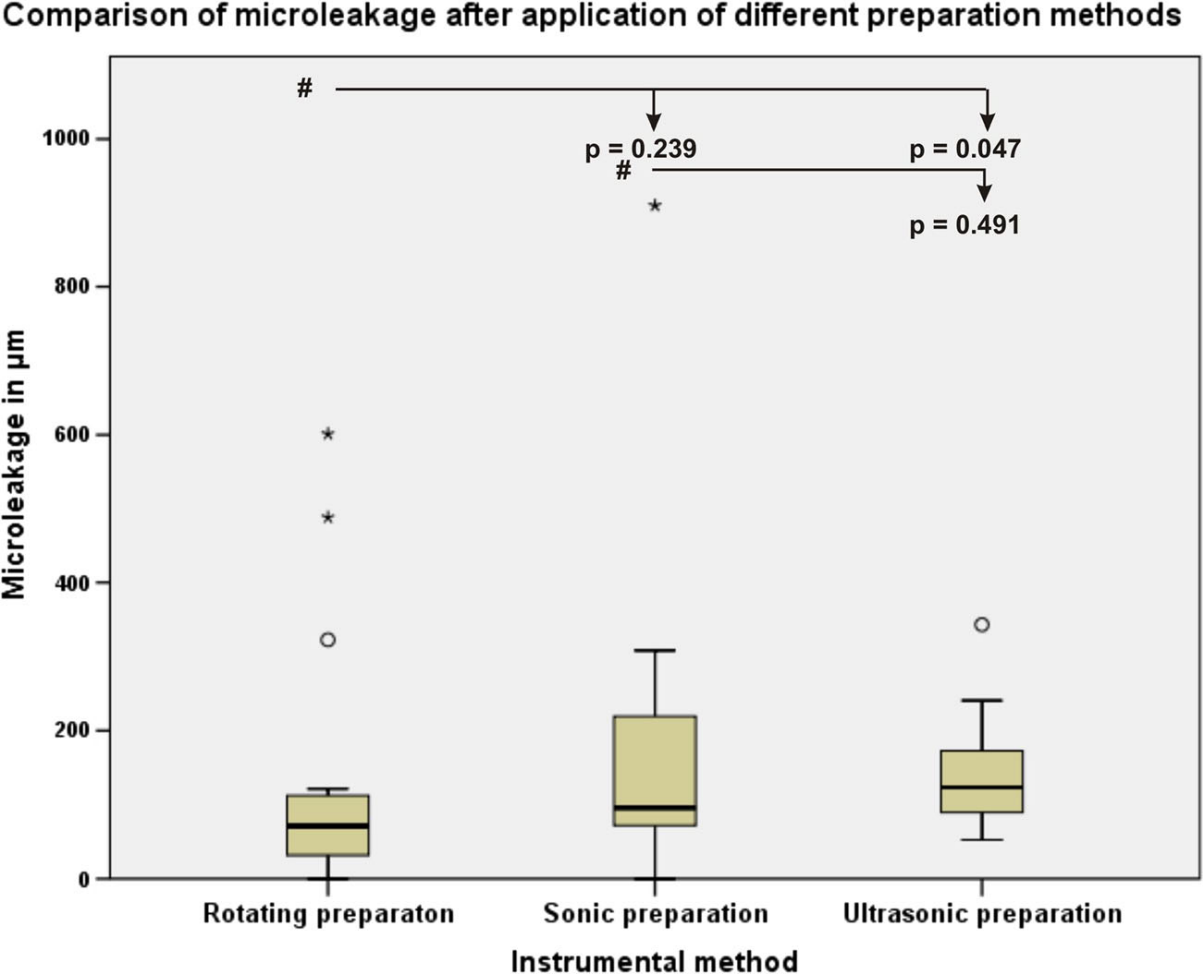
Boxplot of the data distribution of the ceramic inlay proximal microleakage measurements. The pound symbol indicates the comparisons among data

No statistically significant differences were found between any of the groups regarding the ceramic inlay proximal microleakage (Fig. 7).

### Comparison of the ceramic inlay proximal marginal gap after using different proximal box finishing methods

**Fig. 8:**
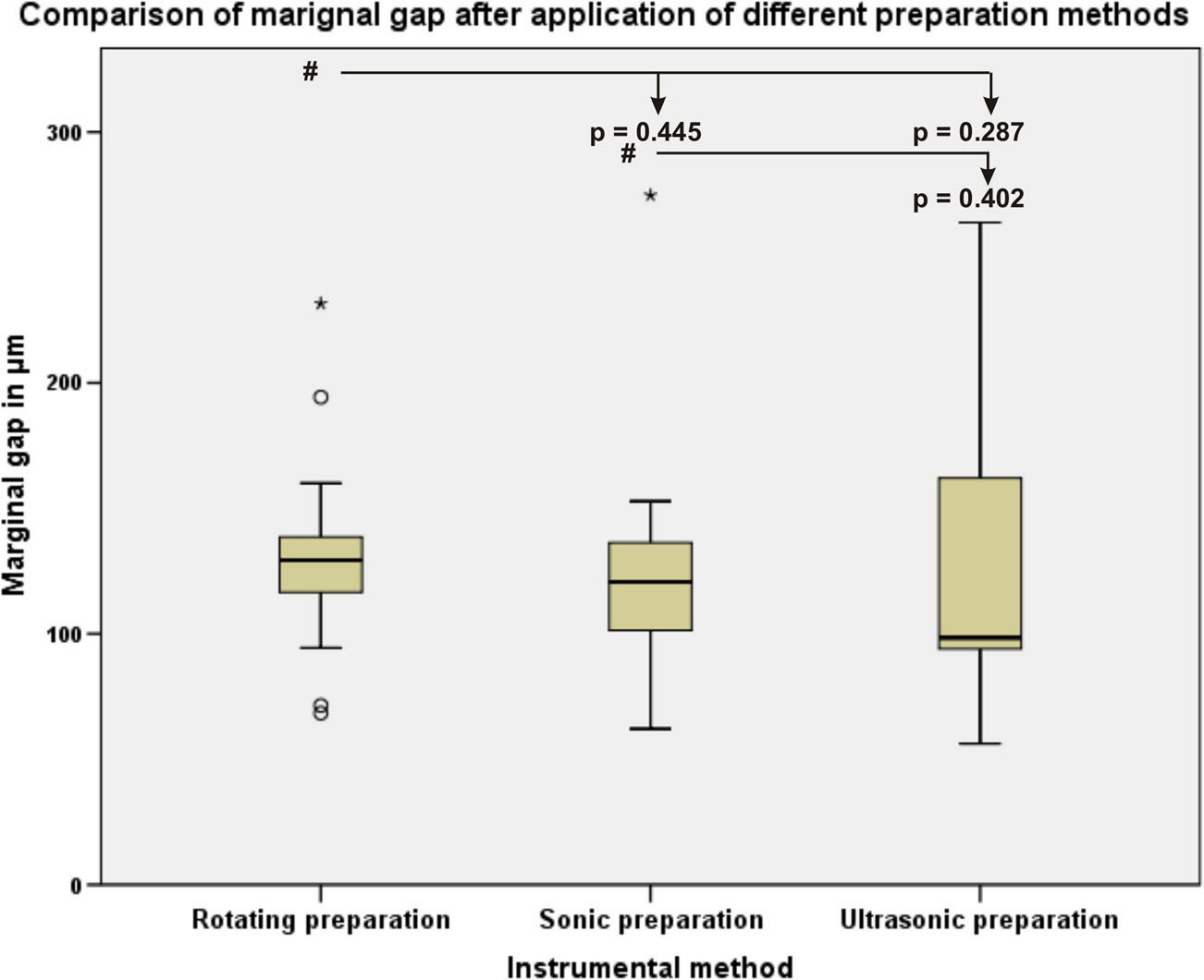
Boxplot of the data distribution of the ceramic inlay proximal marginal gap measurements after using different proximal box finishing methods. The pound symbol indicates the comparisons among data

No statistically significant differences were found between any of the groups regarding proximal marginal gap (Fig. 8).

### Comparison of the ceramic inlay proximal absolute marginal gaps after using different proximal box finishing methods

**Fig. 9:**
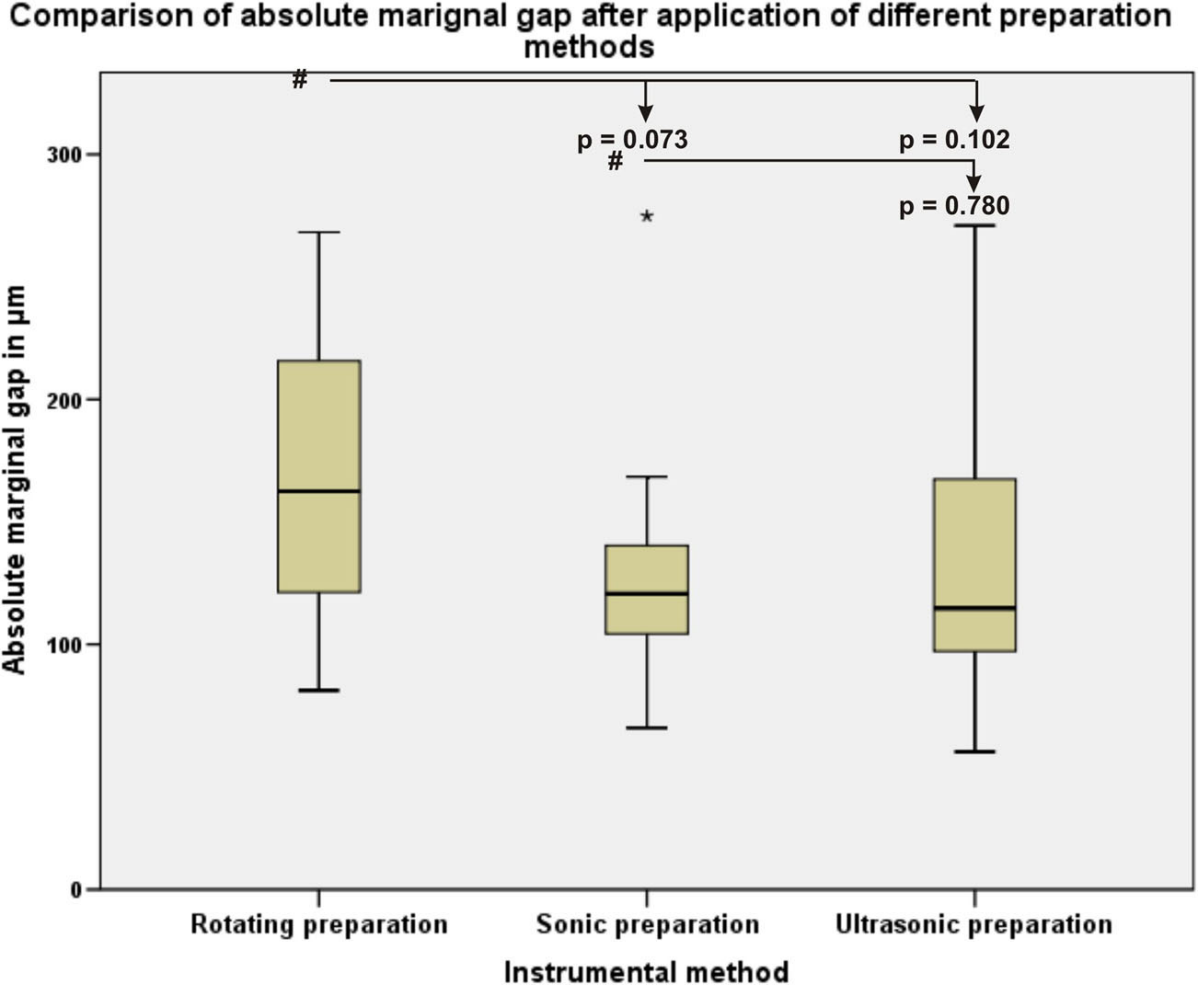
Boxplot graphics of the data distribution of the ceramic inlay proximal absolute marginal gap measurements after using different proximal box finishing methods. The pound symbol indicates the comparisons among data

No statistically significant differences were found between any of the groups regarding absolute marginal gap (Fig. 9).

### Correlation between microleakage, marginal, and absolute marginal gaps

No statistically significant correlations were found between microleakage and marginal gap (*r* = − 0.103; *p* = 0.704), microleakage and absolute marginal gap (*r* = − 0.024; *p* = 0.931), or microleakage and roughness (*r* = − 0.103; *p* = 0.704) in all three groups.

### Quality of the ceramic inlay proximal margin after using different proximal box finishing methods

**Fig. 10:**
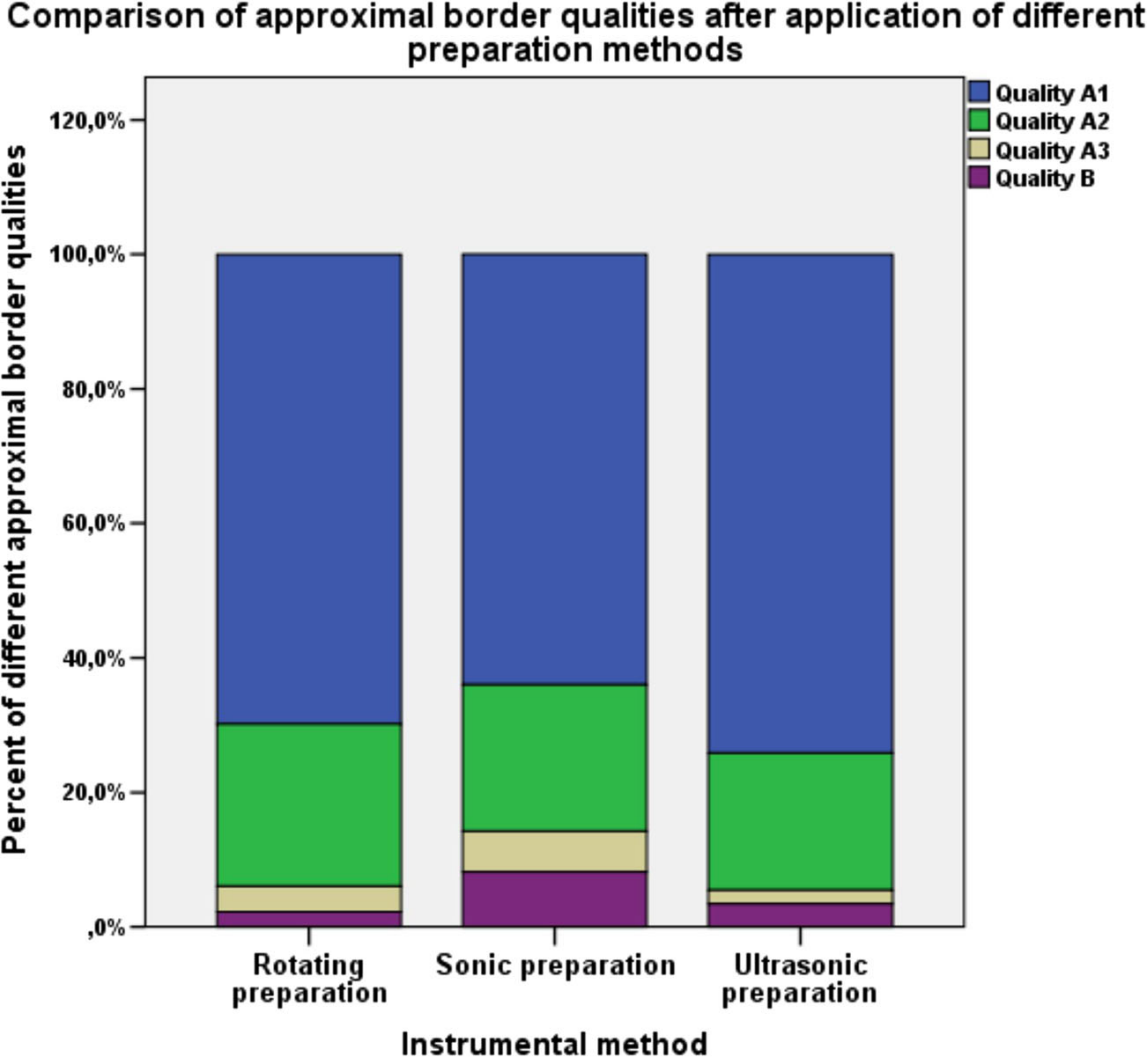
Different ceramic inlay approximal margin qualities after the use of three different finishing methods for the mod-cavity proximal boxes

The distribution of the defined qualities at the ceramic inlay proximal margin was similar in all three groups. Qualities A1 and A2 were most common, and qualities A3 and B were the least common (Fig. 10).

## Discussion

The strategies for prolonging the clinical lifetime of esthetic tooth-colored ceramic restorations are timely [21]. A simple, convenient, and safe way to achieve adjacent tooth preparation (finishing) is a major point of interest for clinicians. Different instruments must be used to stabilize and improve the adhesive interface and the overall dental restoration quality [7]. However, their effects on the dentin and enamel surfaces are contradictory [16]. It is known that rotating instruments, compared to sonic and ultrasonic instruments, result in more mod-cavity proximal box border defects, very irregular surfaces [9], and iatrogenic damage to adjacent teeth**.** Clinically, changes in the marginal quality of ceramic inlays are important because aging (wear) may influence bacterial plaque retention.

Thus, the aim of this in vitro study was to evaluate the marginal quality of the ceramic inlays in human molars after the use of three different instrumental finishing methods. Rotating, sonic, and ultrasonic instruments were used for mod-cavity proximal box finishing in vitro, and their effects on the proximal box floor surface roughness, percentage of continuous margin quality (% of total proximal margin length), microleakage, marginal and absolute marginal gaps after hydrothermal loading (HTL) were assessed. The samples were analyzed via scanning electron microscopy (SEM) and surface roughness analysis.

The data in this study showed a significant increase (*p* < 0.001) in the surface roughness of the proximal box floor after finishing with the sonic and ultrasonic instruments compared to finishing with the rotating instrument. The quality of the dentin surface has influenced the bond strength of the restauration [30]. Differences in the quality of dentin surface and the bond strength after preparation with different kinds of ultrasonic preparation tips have also been reported [16].

The data of this study also clearly demonstrated that microleakage, marginal, absolute marginal gaps after HTL and proximal marginal quality were not significantly different (*p* > 0.05) among the three different instrumental finishing methods. No correlations between microleakage, marginal and absolute marginal gaps or between microleakage and surface roughness were found. This is in accordance with the results of Ellis et al. (2012) that the use of ultrasonic instruments for dentin preparation resulted in bond strengths that were comparable to those obtained with the use of diamond burs [16, 28]. Zaruba et al. (2014) concluded that the preparation design of mod-cavities for ceramic inlays does not influence marginal adaption [31]. Hopp et al. (2013) summarized in their review that the preparation design considerations, fabrication methods, and material choice of ceramic inlays do not influence tooth wear [32]. During the analysis of the finishing protocols, some peculiarities for different instrumental methods were determined. The finishing procedure with the sonic and ultrasonic instruments was more simple and convenient for the operator because finishing was performed without the help of the steel-band matrices and was safe for the adjustment of teeth because of the grainless tips.

The limitations of this study are that artificial aging was performed only with HTL, the qualitative lower proximal marginal gap was only evaluated by one investigator, and this evaluation was not compared with the evaluation of another investigator.

Proximal box finishing of mod-cavities in human molars with SI or USI resulted in a higher value of surface roughness than with RI as a result of the coarser grid of the SI and USI. The type of finishing method did not significantly influence microleakage, marginal or absolute marginal gaps, or proximal box marginal quality.

The data showed that SI and USI increased the surface roughness of the proximal boxes and did not increase microleakage or marginal discrepancy of the ceramic inlays. Therefore, the inlay internal fit, and the proximal marginal quality after the proximal box finishing with SI and USI corresponded to the proximal marginal quality after finishing with RI.

The null hypothesis for the present study, that three different finishing methods of the mod-cavities in human molars will not differ in their influence on the surface roughness and border quality of the proximal boxes, microleakage, and marginal gap of the ceramic inlays within enamel, was partly confirmed.

For the proximal box mod-cavity finishing, the use of SI and USI could be an alternative instrumental method to conventional RI methods, with a lower risk of iatrogenic damage of the adjacent teeth. Clinical research is needed to confirm these findings.

## Conclusion

Ceramic inlays inserted in mod-cavities with proximal boxes finished with SI und USI result in margins of the same quality as those of inlays placed in the mod-cavity after conventional finishing with RI. The advantage of SI und USI is the reduction in the risk of damaging neighboring teeth in comparison to finishing with RI. Therefore, SI and USI are suitable and harmless to neighboring teeth while proximal boxes are being finished. Finishing with SI and USI for mod ceramic inlays results in a marginal quality that are equal to those of conventional mod ceramic inlay finishing with RI.
